# Analysis of risk factors for weaning failure from mechanical ventilation in critically ill older patients with coronavirus disease 2019

**DOI:** 10.1016/j.heliyon.2024.e32835

**Published:** 2024-06-10

**Authors:** Feifan Zhao, Meng Wang, Qingtao Zhou, Yipeng Du, Qin Cheng, Xiaoyan Sun, Jing Zhang, Ying Liang, Ning Shen, Yongchang Sun

**Affiliations:** Department of Respiratory and Critical Care Medicine, Peking University Third Hospital, Beijing, 100191, China

**Keywords:** Coronavirus disease 2019, Invasive mechanical ventilation, Weaning, Diabetes, Older patients

## Abstract

**Objective:**

This study aimed to investigate the factors influencing weaning failure from invasive mechanical ventilation (IMV) in critically ill older patients with coronavirus disease 2019 (COVID-19).

**Methods:**

We enrolled critically ill older patients with COVID-19 who were admitted to the medical intensive care unit (ICU) and received IMV between December 2022 and June 2023.

**Results:**

We included 68 critically ill older patients with COVID-19 (52 male [76.5 %] and 16 female individuals [23.5 %]). The patients’ median age (interquartile range) was 75.5 (70.3–82.8) years. The median length of ICU stay was 11.5 (7.0–17.8) days; 34 cases (50.0 %) were successfully weaned from IMV. The successfully weaned group had a higher proportion of underlying chronic obstructive pulmonary disease [6 (17.6 %) vs. 0, P = 0.033] and fewer cases of diabetes [7 (20.6 %) vs. 16 (47.1 %), P = 0.021] compared with the weaning failure group. Serum lactate levels [1.5 (1.2–2.3) vs. 2.6 (1.9–3.1) mmol/L, P < 0.001], blood urea nitrogen [8.2 (6.3–14.4) vs. 11.4 (8.0–21.3) mmol/L, P = 0.033], Acute Physiology and Chronic Health Evaluation (APACHE) II score [19.0 (12.0–23.3) vs. 22.5 (16.0–29.3), P = 0.014], and hospitalization days before endotracheal intubation [1.0 (0.0–5.0) vs. 3.0 (0.0–11.0), P = 0.023] were significantly decreased in the successfully weaned group, whereas PaO_2_/FiO_2_ [148.3 (94.6–200.3) vs. 101.1 (67.0–165.1), P = 0.038] and blood lymphocyte levels [0.6 (0.4–1.0) vs. 0.5 (0.2–0.6) 10^9^/L, P = 0.048] were significantly increased, compared with the weaning failure group. Multivariate logistic regression analysis showed that diabetes (OR= 3.413, 95 %CI 1.029−11.326), P = 0.045), APACHE II Score (OR = 1.089, 95 % CI 1.008−1.175), P = 0.030), and hospitalization days before endotracheal intubation (OR = 1.137, 95 % CI 1.023−1.264), P = 0.017) were independent risk factors for weaning failure.

**Conclusion:**

In critically ill older patients with COVID-19 with diabetes, higher APACHE II Score, and longer hospitalization days before endotracheal intubation, weaning from IMV was more challenging. The study could help develop strategies for improving COVID-19 treatment.

## 1 Introduction

Approximately 5 % of patients with coronavirus disease 2019 (COVID-19) are severely ill and treated in the intensive care unit (ICU) [1]. The case fatality rate in patients with severe COVID-19 admitted to the ICU is 40–50 % [2], indicating a very high risk of death. Older adults are at a high risk of severe COVID-19, with a significantly increased mortality rate because the risk of severe COVID-19 increases with age [3,4]. China has a large population of older adults; most patients with severe COVID-19 admitted to the ICU are older adults; this is a grim situation, and more attention needs to be paid to the health status and preventive measures in older adults.

In older patients with COVID-19 receiving invasive mechanical ventilation (IMV), successful weaning from mechanical ventilation significantly determines their clinical outcomes. Although IMV is an essential measure for treating patients with severe COVID-19, its prolonged use can lead to dependence. Therefore, successful weaning is critical for patient recovery. This study aimed to investigate the factors influencing weaning failure from IMV in critically ill older patients with COVID-19.

## 2 Materials and methods

### 2.1 Study design

This retrospective cohort study included older patients with severe COVID-19 admitted to the Medical ICU (MICU) of Peking University Third Hospital between December 2022 and June 2023.

The study was approved by the Peking University Third Hospital Medical Science Research Ethics Committee (2023-007-02).

### 2.2 Study participants

**Fig. 1:**
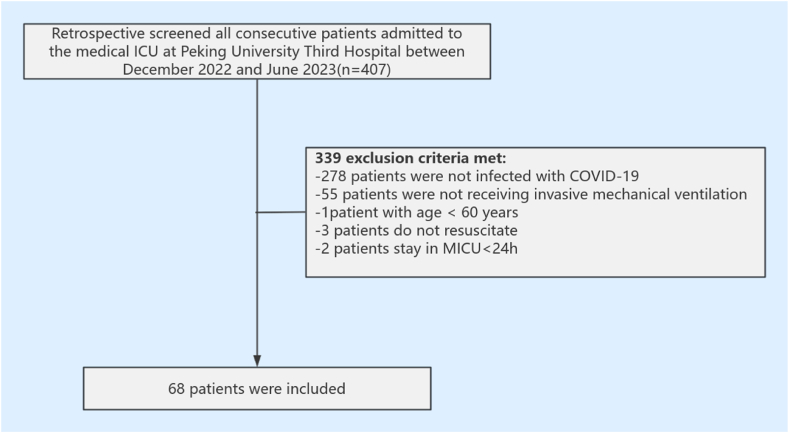
Patient selection flowchart.

The participants were critically ill older patients with COVID-19 who received IMV and were admitted to the hospital's MICU during the study period. The inclusion criteria were: (1) clinical compliance with COVID-19 and positive detection of novel coronavirus nucleic acids and antigens; (2) receiving IMV; and (3) age ≥60 years. The exclusion criteria were: (1) age <60 years, (2) do no resuscitation, and (3) receiving advanced maintenance therapy for malignant tumors. The patient selection flowchart is shown in Fig. 1.

The participants were categorized into successful-weaning and failure-weaning groups based on the weaning outcomes from IMV. Successful weaning was defined as weaning from IMV without reintubation within 72 h. Weaning failure was defined as the failure to wean from the ventilator or reintubation within 72 h after extubation.

### 2.3 IMV weaning protocol

We designed the IMV weaning protocol according to the study by Perkins et al. [5], in which every 2 h, a respiratory therapist assessed a patient for signs of distress or fatigue. In the absence of distress or fatigue, pressure support was reduced by 2 cmH_2_O. This cycle was repeated every 2 h, as tolerated. If, at any point, the patient developed signs of distress or fatigue, then reversible causes were sought, and corrective treatments were initiated, as appropriate. If this failed to resolve the situation, the level of pressure support was increased by 2 cmH_2_O. Spontaneous breathing trials were repeated daily to assess extubation readiness. This cycle continued until the patient either underwent extubation after a successful spontaneous breathing trial or a tracheostomy.

### 2.4 Blood-glucose control protocol

In patients in the ICU, the blood-glucose control protocol was performed with reference to the sepsis guidelines [6]. We started initiating insulin therapy at a glucose level of ≥180 mg/dL (10 mmol/L) and monitored blood glucose for 1 h; a typical target blood glucose range is 144–180 mg/dL (8−10 mmol/L). Insulin administration was reduced and then discontinued if the blood glucose level dropped below 144 mg/dL (8 mmol/L). The frequency of blood glucose monitoring was reduced to 4 h after blood glucose stabilization [7].

### 2.5 Data collection

Patient demographic and clinical data were collected within 24 h after the MICU stay. The information collected included sex, age, underlying disease, complete blood count, liver function, renal function, PaO_2_/FiO_2_, blood lactate level, C-reactive protein, procalcitonin (PCT), serum inflammatory cytokine levels, sequential organ failure assessment (SOFA) score, Acute Physiology and Chronic Health Evaluation (APACHE) II score, hospitalization days before endotracheal intubation, length of IMV, prone ventilation, length of ICU stay, hospital-acquired infection, and drug treatment.

### 2.6 Statistical analysis

Variables are expressed as absolute numbers and percentages or medians and 25th percentile-75th percentile. Clinical data were compared between the two groups of study participants. Measurement data were compared using the non-parametric Mann–Whitney *U* test, and count data were compared using the χ^2^ test. Variables with P < 0.05 were fitted in the multivariate logistic regression (Back Wald) analysis to clarify the independent influencing factors of weaning failure. All statistical analyses were performed using SPSS 22.0, and statistical significance was set to P < 0.05.

## 3 Results

### 3.1 General overview

This study recruited 68 patients (52 male [76.5 %] and 16 female individuals [23.5 %]) who met the inclusion criteria. The patients had a median age (interquartile range) of 75.5 (70.3–82.8) years and a median length of ICU stay of 11.5 (7–17.8) days. The 28-day mortality was 55.9 %.

The weaning success and failure groups comprised 34 patients, respectively. The two groups showed no significant differences in age, sex ratio, or body mass index (Table 1). The weaning success group had a higher proportion of complicated chronic obstructive pulmonary disease (COPD) and a lower proportion of diabetes than did the weaning failure group. Compared with the weaning failure group, the weaning success group had significantly decreased serum lactate level, blood urea nitrogen, APACHE II score, and hospitalization days before endotracheal intubation and significantly higher PaO_2_/FiO_2_ and blood lymphocyte count [0.6 (0.4–1.0) vs. 0.5 (0.2–0.6) × 10^9^ L, P = 0.048] (Table 1). Both groups showed no significant differences in blood leukocytes, neutrophils, platelets, C-reactive protein, PCT, serum creatinine, total bilirubin, or albumin levels (Table 1). The 28-day mortality was 14.7 % and 97.1 % in the weaning success and failure groups, respectively.

**Table 1:** Comparison of demographic and clinical data of the successful weaning and weaning failure groups.

	Successful weaning group (n = 34)	Weaning failure group (n = 34)	Statistic	P-value
**Age** (years)	72.0 (69.8–82.0)	77.5 (71.0–84.3)	Z = −1.253	0.210
**Male sex**	24 (70.6)	28 (82.4)	χ^2^ = 1.308	0.253
**Body mass index** (kg/m^2^)	23.4 (19.6–27.0)	24.5 (21.5–27.2)	Z = −1.202	0.229
**Complication**				
COPD	6 (17.6)	0	χ^2^ = 4.570	0.033
Diabetes	7 (20.6)	16 (47.1)	χ^2^ = 5.322	0.021
Hypertension	21 (61.8)	24 (70.6)	χ^2^ = 0.591	0.442
Cerebrovascular disease	10 (29.4)	10 (29.4)	χ^2^ = 0.000	1.000
Hepatic disease	3 (8.8)	2 (5.8)	χ^2^ = 0.000	1.000
Congestive heart failure	5 (14.7)	0	χ^2^ = 3.454	0.063
Chronic kidney failure	8 (23.5)	8 (23.5)	χ^2^ = 0.000	1.000
**Lactic acid level** (mmol/L)	1.5 (1.2–2.3)	2.6 (1.9–3.1)	Z = −3.842	<0.001
**PaO**_**2**_**/FiO**_**2**_ (mmHg)	148.3 (94.6–200.3)	101.1 (67.0–165.1)	Z = −2.073	0.038
**Serum creatinine** (μmol/L)	77.0 (55.0–104.0)	86.5 (62.5–168.5)	Z = −1.472	0.141
**Serum blood urea nitrogen** (mmol/L)	8.2 (6.3–14.4)	11.4 (8.0–21.3)	Z = −2.134	0.033
**Total bilirubin** (μmol/mL)	11.5 (8.0–14.9)	12.5 (8.8–16.4)	Z = −0.797	0.425
**Albumin** (g/L)	29.6 (27.2–33.6)	31.4 (28.2–33.2)	Z = −0.779	0.436
**Platelet platelets** (10^9^/L)	152.0 (124.8–236.0)	175.0 (99.0–228.3)	Z = −0.049	0.961
**White blood cells** (10^9^/L)	10.3 (7.8–12.1)	10.9 (6.7–14.7)	Z = −0.687	0.492
**Neutrophils** (10^9^/L)	8.6 (6.5–11.3)	9.7 (5.2–13.6)	Z = −0.736	0.462
**Lymphocytes** (10^9^/L)	0.6 (0.4–1.0)	0.5 (0.2–0.6)	Z = −1.975	0.048
**C-reactive protein** (mg/L)	25.5 (8.3–106.3)	27.1 (9.8–71.4)	Z = −0.131	0.896
**Procalcitonin** (ng/mL)	0.3 (0.1–2.3)	0.2 (0.2–1.1)	Z = −0.113	0.910
**SOFA**	8.0 (5.6–9.3)	8.0 (5.8–11.2)	Z = −0.674	0.501
**APACHE II**	19.0 (12.0–23.3)	22.5 (16.0–29.3)	Z = −2.469	0.014
**Hospital-acquired infection**	9 (26.5)	13 (38.2)	χ^2^ = 1.075	0.300
**Days of hospitalization before endotracheal intubation** (days)	1.0 (0.0–5.0)	3 (0.0–11.0)	Z = −2.281	0.023
**Time of mechanical ventilation** (days)	6.0 (5.0–12.5)	7.5 (4.8–12.3)	Z = −0.086	0.931

Data are expressed as n (%) or median (25th percentile −75th percentile).

SOFA: Sequential organ failure assessment; APACHE: Acute physiology and chronic health evaluation; COPD: Chronic obstructive pulmonary disease.

### 3.2 Serum levels of inflammatory factors

In critically ill patients with COVID-19, we collected serum inflammatory factor test data after their stay in the ICU. Although the interleukin (IL)-6 and IL-8 levels increased apparently, no significant differences were observed between the successful and failed weaning groups (P > 0.05) (Table 2).

**Table 2:** Comparison of serum inflammatory cytokine levels.

	Successful weaning group (n = 34)	Weaning failure group (n = 34)	Statistic	P-value
**IL-1β** (pg/mL)	2.5 (1.8–2.9)	2.4 (1.8–3.0)	Z = −0.192	0.848
**IL-2** (pg/mL)	2.5 (1.9–2.7)	2.1 (1.8–2.5)	Z = −0.784	0.433
**IL-4** (pg/mL)	2.5 (1.8–2.9)	2.1 (1.4–2.5)	Z = −1.593	0.111
**IL-5** (pg/mL)	2.0 (1.3–2.5)	1.8 (1.4–2.2)	Z = −0.667	0.505
**IL-6** (pg/mL)	105.4 (42.3–407.8)	65.0 (35.4–470.6)	Z = −0.223	0.823
**IL-8** (pg/mL)	27.7 (14.5–60.1)	41.0 (25.7–81.0)	Z = −1.915	0.055
**IL-10** (pg/mL)	5.3 (4.1–10.2)	7.8 (4.5–18.5)	Z = −1.365	0.172
**IL-12P70** (pg/mL)	2.7 (2.1–3.3)	2.3 (1.9–2.9)	Z = −1.374	0.170
**IL-17** (pg/mL)	7.8 (4.8–10.0)	5.8 (3.3–9.7)	Z = −1.152	0.249
**IFN-α** (pg/mL)	2.5 (1.7–2.9)	2.1 (1.6–2.5)	Z = −1.176	0.240
**IFN-β** (pg/mL)	2.5 (1.6–2.9)	2.2 (1.8–3.1)	Z = −0.399	0.690
**TNF-α** (pg/mL)	2.5 (1.8–2.7)	2.0 (1.5–2.5)	Z = −1.450	0.147

Data are expressed as medians (25th percentile-75th percentile).

IL, interleukin; IFN: Interferon; TNF: Tumor necrosis factor.

### 3.3 Clinical treatment measures

In total, 68 critically ill older patients with COVID-19 were treated following the Chinese Diagnosis and Treatment Guidelines; 17 (25 %) used vasopressors on admission to the ICU, 16 (23.5 %) used nirmatrelvir/ritonavir, 18 (26.5 %) used azvudine, 60 (88.2 %) used glucocorticoid, 26 (38.2 %) used Baricitinib, 32 (47.1 %) used tocilizumab, 61 (89.7 %) received anticoagulant therapy, and 44 (64.7 %) received prone position ventilation. No significant differences were observed between the successful and failed weaning groups (P > 0.05) (Table 3).

**Table 3:** Comparison of clinical treatment measures.

	Successful weaning group (n = 34)	Weaning failure group (n = 34)	Statistic	P-value
**Use vasopressors when entering the ICU**	10 (29.4)	7 (20.6)	χ^2^ = 0.706	0.401
**Glucocorticoid**	31 (91.2)	29 (85.3)	χ^2^ = 0.142	0.707
**Baricitinib**	10 (29.4)	16 (47.1)	χ^2^ = 2.242	0.134
**Tocilizumab**	14 (41.2)	18 (52.9)	χ^2^ = 0.944	0.331
A**nticoagulant therapy**	32 (94.1)	29 (85.3)	χ^2^ = 0.637	0.425
N**irmatrelvir/** **ritonavir**	8 (23.5)	8 (23.5)	χ^2^ = 0.000	1.000
**Azvudine**	9 (26.5)	9 (26.5)	χ^2^ = 0.000	1.000
**Prone position ventilation**	20 (58.8)	24 (70.6)	χ^2^ = 1.030	0.310

Data are expressed as n (%).

ICU: intensive care unit.

### 3.4 Analysis of the predictive factors affecting weaning failure

**Fig. 2:**
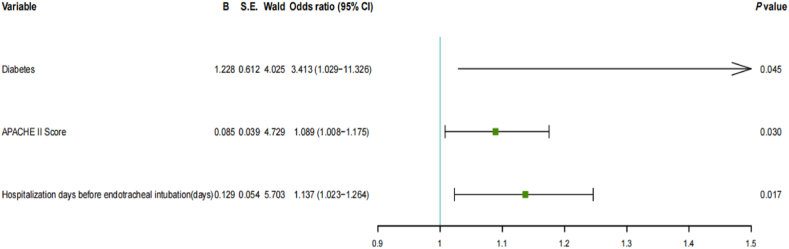
Forest plot of the factors affecting the weaning failure.

Variables with significant differences between the two groups at P < 0.05 were fitted in the multivariate logistic regression (backwalk) analysis to clarify whether they were independent influencing factors of weaning failure. These variables were comorbid COPD, diabetes, serum lactate, APACHE II score, hospitalization day before endotracheal intubation, PaO_2_/FiO_2,_ and hemolymph cell count. Comorbid diabetes, APACHE II score, and hospitalization days before endotracheal intubation were independent influencing factors for weaning failure (Fig. 2).

## 4 Discussion

This retrospective study investigated the clinical characteristics and factors affecting weaning failure in critically ill older patients with COVID-19 receiving IMV. Analyzing the general baseline data of the patients, various factors were considered, including age, sex, basal comorbidities, SOFA score, APACHE II score, PaO_2_/FiO_2_, complete blood count, liver and kidney work, PCT, C-reactive protein, and serum levels of inflammatory factors. In addition, statistical analyses of hospitalization days before endotracheal intubation, prone position ventilation, and treatment measures were performed.

Logistic regression analysis revealed that diabetes, APACHE II score, and prolonged hospitalization before endotracheal intubation were independent risk factors for weaning failure; this suggests that the affected patients were more likely to experience weaning failure from IMV. This means that patients with diabetes, higher APACHE II scores, and longer hospitalization days before endotracheal intubation were more likely to experience weaning failure from IMV.

In a similar study, 50 % of the patients with COVID-19 had associated underlying diseases, such as diabetes, hypertension, and cardiovascular and cerebrovascular diseases [8]. Research has also demonstrated that 32 % of novel coronavirus infections were associated with other diseases, including diabetes (20 %), hypertension (15 %), and cardiovascular diseases (15 %) [9]. In the present study, COPD was more common in the success group. As a result, we thought that patients could have experienced acute exacerbations of COPD rather than hypoxemic pneumopathy, thereby they had a better prognosis. Similar considerations apply to congestive heart failure, which may be an alternative diagnosis of COVID-19 pneumopathy. Therefore, patients with primary onset congestive heart failure may be weaned from IMV more easily once their condition improves. The high risk and poor prognosis of COVID-19 in patients with diabetes are risk factors for weaning failure. In a study including 72,314 patients with COVID-19 in China, the total fatality rate was 2.3 %. However, older patients had a higher fatality rate of 8.0 % in those aged 70−79 years and 14.8 % in those aged ≥80 years. The fatality rate in patients with complications was significantly higher (approximately 7.3 % in older patients with diabetes) [10]. Patients with diabetes had a greater tendency to experience the severe form of COVID-19 than from the moderate level, indicating that the severity of the disease was affected by diabetes [11]. Patients with diabetes had a significantly increased risk of ICU admission, renal failure, and fatality rate after contracting COVID-19 [12]. In the present study, diabetes significantly increased in the weaning failure group. Multivariate analysis indicated that diabetes was an independent risk factor for weaning failure, which is consistent with that in previous studies. Diabetes affects COVID-19 severity and is a critical factor for poor prognosis, particularly in older patients. Therefore, its management is needed to reduce the fatality rate after COVID-19 infection. Regarding medical workers, early identification of COVID-19 in patients with diabetes and implementation of corresponding treatment measures are crucial for improving the survival rate of these patients. Patients with diabetes should be managed through multidisciplinary teamwork and patient-centered management [13]. Due to the ongoing outbreak of COVID-19, research and practice in this area are very significant.

Glucocorticoids should be used with caution for managing patients with severe COVID-19 and diabetes. Patients with diabetes have impaired innate immunity, chronic inflammatory responses, increased coagulation activity, and decreased angiotensin-converting enzyme II expression, wherein using renin-angiotensin-aldosterone system antagonists results in a poor COVID-19 prognosis. Exploring the effects of COVID-19 on patients with diabetes, we found that the novel coronavirus damages islet β cells, leading to reduced insulin secretion, triggering cytokine-induced insulin resistance, and making blood glucose control more difficult. Similarly, drugs used to treat COVID-19 (corticosteroids and lopinavir/ritonavir) may make controlling blood glucose levels in patients with diabetes difficult, leading to a vicious cycle [14,15]. Based on these factors, China's COVID-19 Suggestions for Glucocorticoid Use [16] clearly indicate that in patients with diabetes receiving oral hypoglycemic agents or insulin treatment, glucocorticoid use should be carefully considered when treating COVID-19. Glucocorticoids have anti-inflammatory and anti-allergic effects. However, excessive use may lead to uncontrolled blood sugar, fractures, decreased immunity, and other side effects, which negatively impact patient recovery. Acute hyperglycemia can significantly alter innate immune responses to infection, potentially explaining some poor outcomes in patients who develop hyperglycemia while hospitalized [17]. This leads to the question of how to provide effective treatment in patients with COVID-19 and diabetes while avoiding the risks associated with glucocorticoid use. The development of new drugs, such as tozumab, offers new possibilities. These drugs have anti-inflammatory effects, effectively control the inflammatory storm, and reduce the required glucocorticoid doses. Monitoring patient's blood glucose level, adjusting the hypoglycemic treatment plan as required, and avoiding large fluctuations in blood glucose are also crucial [18]. In the face of the interwoven challenges of COVID-19 and diabetes, understanding patient's condition, drug, and possible side effects; following professional recommendations; and using glucocorticosteroids carefully are crucial. The risk of complications can be reduced by actively looking for alternatives, closely monitoring blood glucose levels, and adjusting treatment plans.

The length of hospitalization before intubation was an independent risk factor for weaning failure. In those with severe COVID-19 who require respiratory therapy, the strength of respiratory support gradually increases as treatment progresses. Oxygen therapy ranges between basic ordinary oxygen therapy and high-flow nasal cannula (HFNC) oxygen therapy, non-invasive ventilation(NIV), or IMV. HFNC and NIV have recently been used in treating acute respiratory failure. However, choosing the appropriate timing for endotracheal intubation is crucial for severely ill patients. Failure of HFNC might cause delayed intubation and worse clinical outcomes in patients with respiratory failure [19]. Therefore, in clinical practice, we need to timely predict the failure of HFNC to reduce the impact of delayed intubation. Older age, higher white blood cell count, higher heart rate, and lower respiratory oxygenation index after HFNC initiation were associated with an increased risk of HFNC failure [20]. Other researchers have considered the possibility of oxygen toxicity as an undesirable feature of HFNC therapy [21]. An intensive study including patients with severe COVID-19 demonstrated that patients with early intubation (within 24 h of ICU entry) had significantly lower mortality rates, fatality rates during hospitalization, and 90-day mortality in the ICU than did those in the delayed intubation group. If the time of early intubation is defined as within 48 h of ICU admission, the results were the same [22]. Furthermore, patients who received HFNC before intubation had lower fatality rates than did those who received NIV. Early or late intubation did not significantly affect all-cause mortality in patients with severe COVID-19. However, other possible interfering factors, such as tight medical resources, need to be excluded [23].

The present study demonstrated that the hospitalization before intubation was significantly shorter in the successful-weaning group than in the failure-weaning group. Furthermore, multivariate analysis indicated that prolonged pre-intubation was an independent risk factor for weaning failure. This observation suggests that timely endotracheal intubation and IMV can improve patients’ prognosis and the weaning success rate from IMV. Therefore, in older patients with severe COVID-19, timely endotracheal intubation, IMV, and minimized pre-intubation hospitalization may contribute to successful weaning from IMV and improve patient outcomes. In clinical practice, physicians should accurately determine the best time for endotracheal intubation based on the disease severity to reduce the risk of weaning failure and improve the survival rate of patients. The advantages and disadvantages of various respiratory support measures to provide a scientific basis for clinical treatment warrant further study.

The relationship between illness severity and the weaning success in patients receiving IMV is crucial in ICU treatment. The risk of weaning failure increases with disease severity. The higher the disease severity was, the more serious the physiological damage to the patient was, leading to a worse response to treatment, which makes weaning patient from IMV difficult. The APACHE II score is vital for assessing disease severity. The scoring system integrates multiple physiological and pathological indicators; the higher the score was, the more severe the disease and the higher the risk of death was [24]. The widespread application of this scoring system in the ICU provides a quantitative assessment of healthcare professionals to guide treatment decisions and prognostic assessments. In the present study, the APACHE II score of the successful-weaning group was significantly lower than that in the weaning failure group. Multivariate logistic regression analysis for predicting weaning failure was also conducted. Our study suggests that close attention should be paid to treating patients with higher APACHE II scores in clinical practice to reduce the risk of weaning failure. We also recommend that various assessment tools be used when assessing illness severity to more accurately reflect the real condition of patients and provide strong support for treatment decisions [25]. APACHE II score is currently the most commonly used disease severity scoring system in ICUs worldwide. During the first 24 h after patient admission to the hospital, the total score is calculated from 0 to 71. The higher the score was, the higher the risk of death was [26]. In future studies, exploring the role of the APACHE II score in other factors predicting weaning failure to provide more valuable references for clinical practice will be necessary.

The weaning process has several risks, particularly in patients with severe diabetes. In the present cohort, diabetes and high APACHE II scores were independent risk factors for weaning failure. The medical team must adopt a more precise and meticulous strategy. Diabetes, hypertension, and obesity are associated with an increased risk of COVID-19 mortality, independent of other known risk factors, particularly in low-resource settings. Addressing these chronic diseases is essential for global pandemic preparedness and mortality prevention [27]. In patients with diabetes, treatment options need to be individualized to ensure that their blood glucose levels are steadily maintained and possible fluctuations are prevented. In addition, other physiological indicators, such as blood lipids and pressure, also need to be closely monitored to ensure the overall health status of patients. Accurately determining the timing of endotracheal intubation is vital to reducing the risk of weaning failure from IMV. When a patient shows signs of respiratory failure, tracheal intubation should be performed promptly to ensure the respiratory tract's patency. During intubation, attention should be paid to patient's vital signs to avoid severe complications, such as cardiac arrhythmia. IMV is a critical means of supporting patients with respiratory failure. The physician should adjust the ventilator parameters based on the patient's mechanical respiratory parameters to achieve effective IMV. In addition, during treatment, antiviral, anti-infection, anti-inflammatory drugs, immune regulation, and other comprehensive therapies are indispensable. Antiviral drugs should be used for viral infections, antibiotics for bacterial infections, and anti-inflammatory treatments can be achieved using hormones. Immunomodulatory therapy can improve immunity and reduce the risk of complications.

This study has some limitations. First, it was limited to older patients. However, most critically ill patients with COVID-19 are older adults, and China has an increasingly aging society. Second, this study included only 68 patients, which may have led to sample-related bias. Third, Since the underlying comorbidities differed between the two groups, the uneven baseline may have biased the final result. Fourth, it was a retrospective observational study conducted at a single center. Large-scale, multicenter, and randomized controlled trials are still required to obtain more accurate and reliable results.

## 5 Conclusion

In older patients with severe COVID-19, diabetes and long hospitalization days before endotracheal intubation were independent factors influencing weaning failure from IMV. According to the characteristics of critically ill older patients with COVID-19 and diabetes or other severe diseases, implementing treatment plan adjustment, determining the timing of endotracheal intubation, and timely IMV would reduce the risk of weaning failure and improve patients' clinical outcomes. This study's findings help gain insight into the clinical characteristics and factors affecting the weaning failure rate in older patients with severe COVID-19 and provide valuable reference information for clinicians to develop more personalized treatment plans and improve the success rate of weaning from IMV.

## Funding

This study was supported by the Novel Coronavirus Infection Cohort Study (Y75505-08) and the Clinical Cohort Building Project (BYSYDL2021019).

## Data availability statement

The data associated with this study has not been deposited into any publicly available repository. It will, however, be made available upon request.

## CRediT authorship contribution statement

**Feifan Zhao:** Writing – review & editing, Writing – original draft, Software, Resources, Formal analysis. **Meng Wang:** Writing – review & editing, Software, Resources, Formal analysis. **Qingtao Zhou:** Writing – review & editing, Supervision, Methodology, Conceptualization. **Yipeng Du:** Investigation, Formal analysis, Data curation. **Qin Cheng:** Investigation, Formal analysis, Data curation. **Xiaoyan Sun:** Investigation, Formal analysis, Data curation. **Jing Zhang:** Investigation, Formal analysis, Data curation. **Ying Liang:** Investigation, Formal analysis, Data curation. **Ning Shen:** Writing – review & editing, Investigation, Formal analysis. **Yongchang Sun:** Writing – review & editing, Methodology, Investigation.

## Declaration of competing interest

The authors declare that they have no known competing financial interests or personal relationships that could have appeared to influence the work reported in this paper.
